# Health system influences on potentially avoidable hospital admissions by secondary mental health service use: A national ecological study

**DOI:** 10.1177/13558196211036739

**Published:** 2021-08-01

**Authors:** Charlotte Woodhead, Peter Martin, David Osborn, Helen Barratt, Rosalind Raine

**Affiliations:** 1 Lecturer Society and Mental Health, ESRC Centre for Society and Mental Health, Department of Psychological Medicine, King’s College London, UK; 2 Lecturer Applied Statistics, NIHR ARC North Thames, Department of Applied Health Research, 4919 University College London, UK; 3 Professor of Psychiatric Epidemiology, Department of Epidemiology and Applied Clinical Research, Division of Psychiatry, University College London and Camden and Islington NHS Foundation Trust, UK; 4 Senior Clinical Research Associate, NIHR ARC North Thames, Department of Applied Health Research, 4919 University College London, UK; 5 Professor of Health Care Evaluation, NIHR ARC North Thames, Department of Applied Health Research, 4919 University College London, UK

**Keywords:** avoidable hospital admission, health care system, mental health

## Abstract

**Objectives:**

Potentially avoidable hospital admissions (PAAs) are costly to health services and potentially harmful for patients. This study aimed to compare area-level PAA rates among people using and not using secondary mental health services in England and to identify health system features that may influence between-area PAA variation.

**Methods:**

National ecological study using linked English hospital admissions and secondary mental health services data (2016–2018). We calculated two-year average age-sex standardised area-level PAA rates according to primary admission diagnoses for 12 physical conditions, among, first, secondary mental health service users with any non-organic diagnosis, and, second, people not in contact with secondary mental health services. We used penalised regression analyses to identify predictors of area-level variation in PAA rates.

**Results:**

Area-level PAA rates were over four times greater in the mental health group, at 7,594 per 100,000 population compared to 1,819 per 100,000 in the comparator group. Common predictors of variation were greater density of older age groups (lower PAA rates), higher underlying population morbidity of chronic obstructive pulmonary disease and, to a lesser extent, urbanity (higher PAA rates). For both groups, health system factors such as the number of general practitioners per capita or ambulance despatch rates were significant but weak predictors of variation. Mental health diagnosis data were available for half of secondary mental health care records only and sensitivity analyses found that urbanity remained the sole significant predictor for PAAs in this group.

**Conclusions:**

Findings support the need for improved management of physical conditions for secondary mental health service users. Understanding and predicting variation in PAAs among mental health service users is constrained by availability of data on mental health diagnosis, physical health care and needs.

## Introduction

Potentially avoidable admissions (PAAs) refer to admissions for conditions that could be managed outside hospital through appropriate care.^1^ PAAs are costly to health services and may adversely affect patients’ wellbeing. ^2^ High PAA rates are considered indicative of sub-optimal health care system functioning, reflecting poor quality or limited access to primary care and community services.^3^ PAA present a particular challenge for people with mental health problems, particularly those with more severe conditions who more frequently attend emergency departments (ED)^4,5^ and experience higher PAA rates than the general population.^4,6^ This is likely due to greater risk of morbidity or multimorbidity and premature mortality, predominantly linked to physical conditions.^7,8^ People with mental health problems may be at particular risk of incurring harm through unplanned hospital visits and PAA specifically, because ED staff may lack adequate skills to provide appropriate and non-stigmatising care.^9^

In England, reducing PAA rates has been a key policy objective for some time.^10^ Available research has examined reasons for variation in PAAs between general practices, hospitals, or local areas in both the general population and some patient groups.^3^ This found that population deprivation accounts for a large proportion of between-area or practice variation, while wider health system factors such as access to general practice appear to contribute less.^3^ However, deprivation levels are largely outside the control of those purchasing or delivering health and care services, and it is therefore important to identify those health system factors that are associated with PAA and that are amenable to intervention to inform targeting of initiatives to reduce such admissions.

There has been little work on PAA among people with mental health problems and this study seeks to fill this gap by investigating PAAs among adults with psychoses or non-psychotic mental health conditions who have been in contact with secondary mental health services in England. Specifically, we aimed to: (1) compare the physical health conditions for which PAAs occur; (2) estimate and compare PAA rates at the level of Clinical Commissioning Groups (CCGs) (bodies responsible for planning and commissioning health care for local areas in England), and extent of variation between them; and (3) compare health system features predicting variation in PAAs between CCGs, including supply (e.g. health service availability and quality), demand (e.g. population morbidity), and other characteristics (e.g. rurality).

## Methods

We conducted a national-level ecological study examining variation in PAA rates for people with selected mental health problems during 2016 to 2018 at the CCG-level in England.

### Data sources

We used, first, Hospital Episodes Statistics (HES) Admitted Patient Care and ED data containing details of all hospital admissions and ED attendances at English National Health Service (NHS) hospitals.^11^ We requested pseudonymised data on unplanned hospital admissions (emergency due to clinical need) and ED data for two years, 2016/17 and 2017/18. Second, we used the Mental Health Services Dataset (MHSDS), which is a patient-level secondary uses dataset containing information about people in contact with community, outpatient, and hospital mental health services in England.^12^ It includes information about use of NHS-funded specialist and secondary mental health care. MHSDS data for adults were requested for the period April-March during both 2016/17 and 2017/18, the maximum number available due to MHS reporting changes. Linkage between HES and MHSDS is possible via a bridging file which details pseudonymised HES records of those in contact with (all activity relating to patients who receive services) secondary mental health services during this time.

Mental health condition information is available in MHSDS based on primary diagnosis (International Statistical Classification of Diseases, version 10, ICD-10)^13^ and care cluster assignment; a care cluster describes a group of people with similar characteristics (e.g. non-psychotic, psychotic, organic).^14^ Using this information, we identified MHSDS patients with any psychoses or non-psychotic conditions. On average, cluster or diagnosis data were missing for 49.5% of MHSDS patients per CCG, with wide between-CCG variation (21.1% to 97.6%) (please see Online Supplement for further details on information used to categorise patients and missing data).

### Identification of patient groups and potentially avoidable admissions

Within each CCG, we categorised (1) the mental health user group and (2) the comparator. The mental health user group included HES unplanned admission records for adults aged 18 years and over linked to MHSDS (current secondary mental health service users) recorded with any non-organic mental health diagnosis (e.g. schizophrenia, bipolar affective disorder or personality disorder). The comparator group included all HES unplanned admission records (adults aged 18+) not linked to MHSDS during the study period. This group may include people with low-level mental ill health who have been in contact with primary care only or did not require services, as well as those with more serious mental ill health that is managed in primary care.

PAAs were identified from the HES primary diagnosis code for the first finished consultant episode, which is the first continuous period of admitted patient care under one consultant (specialist doctor) within one health care provider in the year of interest. We considered admissions for 12 physical diagnoses for adults to be potentially avoidable (see Online Supplement Table S1).^1,3^

### Calculation of CCG-level potentially avoidable admission rates

Using HES admissions data, we calculated annual average CCG-level PAA rates for adults per 1,00,000 people for each group and year. CCG of admission was identified from ‘CCG of responsibility’ for the patients’ care. Records were excluded if the CCG of responsibility was not in England. Missing data were assigned to the CCG where treated if available, or otherwise coded as missing. We used direct standardisation of the number of PAAs in each group for each CCG (age groups: 18–34, 35–44, 45–54, 65–74, 75–84, 85+ years) and binary sex category to account for different CCG population structures. We calculated mean directly standardised rates per 1,00,000 people per year, using the population of England in 2016 as standard population.

### Predictors of variation in standardised rates of potentially avoidable admissions

We identified putative predictors of variation in PAA rates from the existing literature^3–5^ and data availability at CCG-level (Table 1), capturing indicators in five domains: population socio-demographics and geography; population underlying morbidity; hospital, ED, and ambulance; primary care general practice; and secondary mental health service spending and performance. Mental health system predictors were also considered in comparator group analyses since it included people with mental health problems who were not currently in contact with secondary mental health care.

**Table 1.** Predictors of potentially avoidable hospital admissions and data source.

Predictor	Source^a^
*Socio-demographics and geography*	
Index of Multiple Deprivation 2015. Summary measures of deprivation at CCG-level geography for each of six domains: income; employment; education, skills and training; crime; barriers to housing and services; living environment.	GOV.UK
% of CCG population aged over 75 years	ONS
% of CCG population identifying as Black and minority ethnicities	ONS
Six-point urban/rural classification scale	ONS
Geographical location (North, Midlands or South England)	ONS
*Underlying morbidity*	
2016/17 prevalence of: chronic obstructive pulmonary disease (COPD); diabetes mellitus; hypertension; serious mental illness; depression	NHS Digital
% of population aged 18+ years in contact with mental health services	MHSDS
% of mental health service users treated under the Mental Health Act	NHS Digital
*Hospital, Emergency Department (ED) and ambulance*	
Directly age/sex standardised ED attendance rate	HES
Median referral to treatment time (weeks)	NHS England
% of all unplanned hospital admissions which were referred by GPs	HES
% of ambulance calls with a face-to-face response not transported to major or speciality EDs (Type 1 or Type 2 in the UK) (non-conveyance)	NHS England
% of calls to a national non-emergency telephone line (‘NHS 111’) that were referred to ED	NHS England
% of calls to a national non-emergency telephone line (‘NHS 111’) for which an ambulance is despatched	NHS England
*Primary care general practice*	
% of single-handed GP’s	NHS Digital
% not able to make an appointment to speak to or see someone	GP Patient Survey
% able to see GP/nurse within 48 hours	GP Patient Survey
GP's per 1,00,000 population	NHS Digital
Quality and Outcomes Framework (QoF) achievement rate^b^	NHS Digital
Improving Access to Psychological Therapies (IAPT) access rate^c^	NHS Digital
% waiting more than six weeks for IAPT treatment from referral	NHS Digital
*Secondary mental health service spending and performance*	
% of total core CCG budget allocation spent on mental health services overall	NHS Digital
% of total core CCG budget allocation spent on early intervention in psychosis	NHS Digital
% of total core CCG budget allocation spent on crisis resolution home treatment team	NHS England
% of total core CCG budget allocation spent on ED Liaison services	NHS England
% of mental health service users with a CPA in place followed up within 7 days of leaving psychiatric hospital^d^	NHS England
% of people on CPA in employment^e^	NHS England
% of admissions to psychiatric inpatient wards gate-kept by a CRHT team^f^	NHS England

Note: ONS = Office for National Statistics; NHS = National Health Service; MHSDS = Mental health services dataset; CCG = Clinical Commissioning Group; QOF = Quality and Outcomes Framework; ED = Emergency Department; GP = general practice/practitioner; MH5YFV = Mental Health Five Year Forward View; CPA=Care Programme Approach; CRHT=Crisis resolution home treatment team; PAA=potentially avoidable hospital admission; IAPT=improving access to psychological therapies.

^a^See online supplementary material for source references.

^b^The Quality and Outcomes Framework (QoF) is a voluntary annual reward and incentive programme for all GP surgeries in England.

^c^The Improving Access to Psychological Therapies (IAPT) service provides talking therapies for commonly occurring mental health problems (e.g. depression, anxiety) through primary care, individuals can be referred by their GP, self-refer or be referred by community or secondary health services.

^d^The Care Programme Approach (CPA) is a package of care used to plan mental health care for some people with mental health problems (e.g. those with serious mental health condition, or at risk of suicide or self-harm). People with a CPA in place should be followed up within a week of leaving psychiatric hospital to reduce the risk of suicide and social exclusion and improve care pathways.

^e^The proportion of people with a CPA in place that are recorded as being employed. CPA plans include support with access to employment.

^f^Crisis Resolution and Home Treatment (CHRT) services provide support for people in the community who experience a mental health crisis while out of hospital to help prevent potentially avoidable admissions.

### Statistical analyses

We first conducted descriptive analyses to examine the characteristics of each group. To examine CCG-level PAA rates, we then calculated the median, interquartile range (IQR) and range of standardised PAA rates. To estimate associations of CCG characteristics with PAA rates we used a linear model of the form

$${\mathrm{PAA}}_{i}=\beta_{0}+\sum_{k=1}^{38}\beta_{k}X_{ki}$$

where *PAA_i_* is the standardised PAA rate for the *i*^th^ CCG (*i* = 1, …, 207), and the *X_ki_* represent the potential predictors (Table 1) (see also Online Supplement Table S2).

To estimate the slope coefficients $\beta_{k}$ , we employed a type of penalized linear regression, the ‘lasso’ (least absolute shrinkage and selection operator).^15,16^ Like ordinary least squares (OLS) regression, the lasso finds estimates of regression coefficients by minimising the residual sum of squares. In contrast to OLS regression, the lasso constrains the sum of the absolute values of the coefficients to be less than a constant. This produces smaller (‘shrunk’) coefficients than OLS and tends to result in some coefficient estimates being exactly zero. Compared to OLS, lasso estimates are biased towards zero, but have smaller variance. Compared to other methods of penalized regression, such as ridge regression, the lasso allows for variable selection, which helps with interpretability since covariates not associated with the outcome are removed from the model. The lasso is therefore useful in situations where the number of predictor variables is large relative to the number of cases.

#### Missing values and the multiple imputation random lasso (MIRL)

Seven of the 38 predictor variables had missing values, with an overall number of missing values of 39 (0.5% of all covariate values). Of 207 CCGs, 180 had complete data, others had between one and four missing values. Although the extent of missingness was small, a complete cases analysis would have reduced our dataset and likely have led to bias. We therefore employed multiple imputation of missing values by chained equations^17^ and the multiple imputation random lasso (MIRL)^18^ approach to combine lasso estimates from several imputed datasets. This way, we were able to include all 207 CCGs in our regression analyses (see Online Supplement for further detail). We report the final MIRL standardised coefficient estimates. We conducted sensitivity analyses for CCGs with at least 30% of diagnosis data available (n = 180), and again for CCGs with at least 50% of diagnosis data available (n = 127).

Analyses used STATA v.15(19) and R v. 3.5.0,^20^ including specific R packages: ggplot2, psych, glmnet, mfp, mice.

### Ethical approval

The study received ethical approval from the NHS Health Research Authority, reference 18/HRA/1102. The study used routinely collected anonymised administrative data and did not affect the type of care that patients received. Consent by patients was not required.

## Results

Between April 2016 and March 2018 there were 1,00,42,770 emergency unplanned hospital admissions among over 18 year olds in England, of which 34,68,201 were admissions with a record in MHSDS.

The mental health user group was proportionately more likely to be female, younger, to live in urban and more deprived areas than the comparator group (Online Supplement). The socio-demographic characteristics of those with missing data were similar to those of the average values of those for which diagnosis or cluster data were available. The only exception were female sex, which was slightly less common among those with missing data. People with missing diagnosis data were also proportionately more likely to be flagged with learning disability than those with available data (8.3% vs 4.3%, Online Supplement). This means that some of these patients would have been receiving services for learning disabilities but not mental health services.

### Physical conditions comprising PAAs

Table 2 shows potentially avoidable admissions and the distribution of condition type by mental health status. The most common primary diagnoses among, respectively, the mental health and comparator groups were non-specific chest (21.9% and 26.8%) and abdominal pain (23.7% and 22.1%), followed by chronic obstructive pulmonary disease (COPD) (15.2% and 13.6%) and urinary tract infection (13.7% and 12.6%).

**Table 2.** Potentially avoidable admissions and distribution of condition type by mental health status.

	Potentially avoidable admissions (PAA)	
	Mental health user group^a^(N = 1,16,997)	Comparator group^b^ (N = 12,01,141)
Condition	*n* (%)	*n* (%)
Non-specific chest pain	25,674 (21.9)	3,22,398 (26.8)
Non-specific abdominal pain	27,753 (23.7)	2,64,989 (22.1)
Urinary tract infection	15,985 (13.7)	1,50,959 (12.6)
Chronic obstructive pulmonary disease	17,788 (15.2)	1,62,978 (13.6)
Cellulitis	7246 (6.2)	1,01,604 (8.5)
Fall	6796 (5.8)	56,534 (4.7)
Angina	3151 (2.7)	54,776 (4.6)
Epilepsy	6263 (5.4)	19,410 (1.6)
Deep vein thrombosis	2097 (1.8)	35,924 (3.0)
Blocked urinary catheter	1115 (1.0)	13,303 (1.1)
Hypoglycaemic diabetic episode	1821 (1.6)	11,585 (1.0)
Minor head injury	1308 (1.1)	6681 (0.6)
Total	1,16,997 (100.0)	12,01,141 (100.0)

^a^Records linked to mental health services dataset over the study period with a primary diagnosis or care cluster assignment linked to any non-organic mental health condition (including psychotic and non-psychotic conditions).

^b^Comparator: records not linked to mental health services dataset over the study period.

### CCG level variation in PAA rates

**Figure 1.**
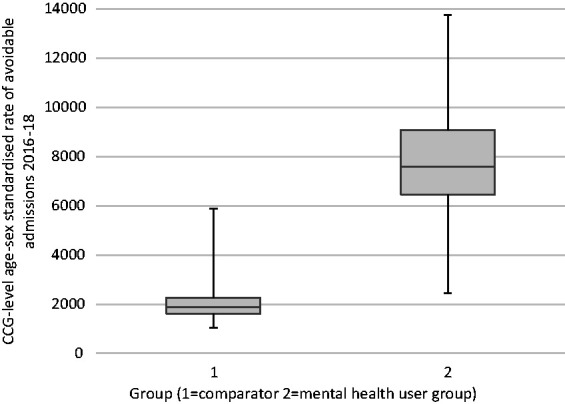
Clinical Commissioning Group-level age-sex directly standardised rate of potentially avoidable hospital admissions† per 1,00,000 population in people with and without contact with secondary care mental health services. Note: Figure shows Median, interquartile range (IQR), and range (minimum-maximum) of CCG values. Whiskers indicate range of CCG values, IQR indicated by grey box. 1. Comparator group: records not linked to mental health services dataset over the study period. 2. Mental health user group: records linked to mental health services dataset over the study period with a primary diagnosis or care cluster assignment linked to any non-organic mental health condition (including psychotic and non-psychotic conditions). †Potentially avoidable admissions defined in relation to a set of 12 physical conditions.

After standardising for age and sex, the CCG-level mean PAA rate was 4.2 times greater in the mental health than the comparator group (7,594 vs 1,819 avoidable admissions per 1,00,000 population), with a 5.6-fold and 6.1-fold difference between CCGs with the lowest and highest PAA rates (Figure 1).

### Predictors of variation in CCG-level PAAs

The most important factors predicting higher PAA rates in the mental health group were: higher prevalence of COPD in the CCG population, a lower proportion of those aged over 75 years, and a lower proportion of adults in contact with secondary mental health services (Table 3). These were followed by the proportion of those receiving psychological therapies treatment (IAPT) within six weeks, the proportion of non-emergency telephone line calls resulting in ambulance despatch, and urbanity. Sensitivity analyses of CCGs with at least 30% of available mental health diagnoses data complete identified the same predictors of PAA rates with very similar point estimates for the coefficients. The only exception was the proportion of non-emergency telephone line calls resulting in ambulance despatch. Further sensitivity analysis considering only CCGs with at least 50% of available mental health diagnoses data complete (n = 127) found only urbanity to be predictive of higher PAA rates in the mental health group.

**Table 3.** Predictors of avoidable admission on CCG characteristics among the mental health user and comparator groups.

Predictor	Estimated standardised coefficient (bootstrap quantiles: 2.5%, 97.5%)	
	Mental health user group^a^	Comparator group^b^
COPD prevalence	0.597 (0.293, 0.908)	0.426 (0.181, 0.687)
Population aged over 75 years (%)	−0.350 (−0.617, 0.000)	−0.468 (−0.739, −0.148)
Adult population in contact with secondary mental health services (%)	−0.320 (−0.481, 0.000)	
Receipt of IAPT treatment within 6 weeks^c^ (%)	0.189 (0.000, 0.305)	
NHS 111 ambulance despatch rate^d^	0.155 (0.000, 0.309)	
Urban	0.111 (0.000, 0.280)	0.093 (0.000, 0.247)
Diabetes prevalence		0.307 (0.000, 0.549)
Region: South		−0.185 (−0.409, 0.000)
Number of GPs per 1,00,000		0.173 (0.000, 0.325)
Ambulance non-conveyance^e^ (%)		0.166 (0.000, 0.361)
Admissions from GP (%)		0.153 (0.000, 0.288)
Admissions to acute wards gatekept by CHRT^f^ (%)		−0.096 (−0.226, 0.000)

Note: N = 207. All coefficients are xy-standardised. Bootstrap quantiles represent the quantiles of the coefficient distribution generated by bootstrapping in the final stage of MIRL. They are not confidence intervals in the strict sense and are given for informal illustration only. CCG=Clinical Commissioning Group; MIRL=multiple imputation random lasso COPD=chronic obstructive pulmonary disease; GP=general practitioner; CRHT=crisis resolution home treatment team; IAPT=improving access to psychological therapies; NHS=National Health Service.

^a^Records linked to mental health services dataset over the study period with a primary diagnosis or care cluster assignment linked to any non-organic mental health condition (including psychotic and non-psychotic conditions).

^b^Comparator: records not linked to mental health services dataset over the study period.

^c^The Improving Access to Psychological Therapies (IAPT) service provides talking therapies for commonly occurring mental health problems (e.g. depression, anxiety) through primary care, individuals can be referred by their GP, self-refer, or be referred by community or secondary health services. There is a 6-week target for accessing treatment following referral.

^d^Proportion of calls to a national non-emergency telephone line calls (‘NHS 111’) for which an ambulance is despatched.

^e^Proportion of ambulance calls with a face-to-face response not transported to major or speciality EDs (Type 1 or Type 2 in the UK) (non-conveyance).

^f^Crisis Resolution and Home Treatment (CHRT) services provide support for people in the community who experience a mental health crisis while out of hospital to help prevent potentially avoidable admissions.

Among the comparator group, the leading predictors of higher PAA rates were a higher COPD and diabetes (types 1 and 2) prevalence and a lower proportion of those aged over 75 years, followed by: non-Southern location, urbanity, a greater number of GPs per 1,00,000 population, a greater proportion of admissions referred by GPs, higher ambulance non-conveyance rates (proportion of ambulance calls with a face-to-face response not transported to Emergency Department), and a smaller proportion of admissions gate-kept by crisis resolution and home treatment (CHRT) teams (CHRT services provide support for people in the community who experience a mental health crisis while out of hospital) (See Online Supplement Table S2).

## Discussion

This study found area-level rates of potentially avoidable hospital admissions (PAAs) in England to be four times greater among secondary mental health service users with any psychotic or non-psychotic mental health condition than people not using secondary mental health services. Selected long-term conditions and older age most strongly predicted PAA rates for both groups, with some impact of selected indicators of access and performance, too.

Our capacity to predict and understand variation in PAA rates was limited by the availability of mental health diagnosis data, with sensitivity analyses leaving urbanity as the sole predictor of (greater) PAAs. We are thus not able to arrive at strong conclusions about the importance of other predictor variables investigated. However, our findings suggest that better management of physical conditions for people in contact with secondary mental health services could reduce PAAs in this group. There is urgent need for better recording of psychiatric diagnosis and data on physical ill health severity, along with quality data on health care performance for those receiving secondary mental health services to further our understanding about factors influencing PAAs and between-area variation.

### Strengths and limitations

Use of population-level routine administrative data permitted national comparison of areas responsible for the planning and purchasing of health services. Unlike previous studies,^4^ we were able to validate presence of mental ill health through linkage with secondary mental health services data. We also examined predictors of area-level variation in PAA rates using a robust analytical approach. This contrasts with an earlier study of the general population,^3^ which used hierarchical stepwise forward regression analyses, now seen as an inappropriate and biased approach to variable selection.^21^

There are several limitations to our study. First, a major weakness is the limited availability of diagnosis data, which were only available for half of mental health records linked to the hospital episode statistics. This potentially introduced bias, particularly for estimations of predictors of area-level variation in PAA rates, with high variability of missing data across CCGs. People with more contacts with mental health services, who are potentially more unwell, will be more likely to have a diagnosis recorded than those receiving community care. This is a well-known limitation of this type of data and we took several steps to explore the effect of missing data on our findings. Second, the comparator group included people receiving mental health support solely in primary care or those who might have previously used specialist mental health services. The implication of this is that observed differences in PAA rates by mental health status may be greater than estimated. At the same time, PAA rates are likely overestimated because not every admission for included conditions will have been avoidable^1^ although this should not differ between CCGs or groups. An alternative indicator of suboptimal care could be potentially preventable readmission although defining ‘preventability’ remains challenging.^22^ Third, we could only include predictors of variation for which CCG-level data were available. We were not able to include other potentially important predictor variables, such as average travel time to hospital or physical health care in mental health services. Also, there are rising and variable thresholds for accessing secondary mental health services across England^23^ which potentially introduced further bias. For instance, areas with lower thresholds likely include a greater proportion of people with less severe mental illness on MHSDS or have less pressure on services, and, by implication, lower PAA rates.

### Interpretation and comparison with existing literature

Mental health and comparator groups displayed similar patterns of physical conditions in relation to PAAs and higher PAA rates in this group have previously been reported.^4,6^ This likely reflects more complex care needs, greater risk of physical conditions and multimorbidity, including substance use.^24,25^ It may also reflect greater physical condition severity, delayed help-seeking, or suboptimal management of physical health in secondary mental health care.^26^ It could also indicate that secondary mental health services are detecting physical ill health and are admitting, signposting or accompanying people directly to acute hospitals. However, appropriate data were not available to assess these possibilities further.

As noted, the strongest predictors of area-level variation in PAA rates were underlying population morbidity and age. Perhaps counterintuitively, a higher proportion of people over the age of 75 years in a CCG predicted lower PAA rates; we included this indicator as a marker of the extent of pressure on services within the wider health care system. Previous work also found a higher proportion of this age group to be associated with lower PAA rates, although this association was no longer significant after adjustment for deprivation.^3^ Avoidable admissions tend to be greater among over 75 s in general,^27^ and it may be that the this indicator reflects other characteristics of CCGs that influence lower PAA rates, for example, rural setting with poorer transport access to hospital. Areas which attract a greater proportion of retired people may also be less deprived.

In contrast to previous general population studies, deprivation did not significantly predict CCG-level variation for either group in our study. This may be partly because previous studies included overall deprivation scores, which include information on, for example, emergency hospital admissions. It is also possible that the mechanisms through which deprivation likely increases PAAs such as higher levels of health risk factors (e.g. smoking, poor diet) were indirectly captured in our models by predictors such as COPD and diabetes prevalence. Also, urban areas tend to be more deprived than rural areas overall and while some of the variance accounted for by urbanity may therefore be linked to deprivation, other unaccounted for factors may be associated with higher PAAs, for example, higher hospital concentration, shorter travel time in urban areas. Similarly, in the comparator group, CCGs located in the south of England had significantly lower PAA rates, which tend to be less deprived on average than those in the north.^28^

Our study was unable to demonstrate strong evidence for the impact of what we considered to be health system features in relation to PAAs. For example, none of the GP care quality measures that we included predicted variation in PAA rates. There was some indication that greater access to primary care may increase PAAs in the comparator group, while in the mental health group, a greater non-emergency telephone call ambulance despatch rate predicted higher PAA rates. Almost a third of people with serious mental health problems in England are treated in primary care,^29^ and it may be that GP access is less influential for people in the mental health group, for whom responsibility for managing physical health is less clear.^26^

Further, differences in thresholds for selection into secondary mental health care may partly explain unanticipated predictors for the mental health group. For instance, a greater proportion of the population in contact with mental health services, which may be indicative of lower thresholds, was associated with lower PAA rates. Similarly, a higher proportion of referrals to psychological treatment services may reflect greater priority given to primary rather than secondary mental health services in selected areas.^30^ Such areas may have higher secondary care access thresholds, selective of more severe mental health problems, thus with higher PAA rates. Further work assessing area-specific selection into mental health services and severity or complexity of underlying conditions is needed to further disentangle this.

## Supplemental Material

sj-pdf-1-hsr-10.1177_13558196211036739 - Supplemental material for Health system influences on potentially avoidable hospital admissions by secondary mental health service use: A national ecological study Click here for additional data file. Supplemental material, sj-pdf-1-hsr-10.1177_13558196211036739 for Health system influences on potentially avoidable hospital admissions by secondary mental health service use: A national ecological study by Charlotte Woodhead, Peter Martin, David Osborn, Helen Barratt and Rosalind Raine in Journal of Health Services Research & Policy
