# Supplementary material for: Health system influences on potentially avoidable hospital admissions by secondary mental health service use: A national ecological study
Source: J Health Serv Res Policy. 2021 Aug 1;27(1):22–30. doi: 10.1177/13558196211036739 (PMC8772012; doi:10.1177/13558196211036739)
Supplement: sj-pdf-1-hsr-10.1177_13558196211036739 - Supplemental material for Health system influences on potentially avoidable hospital admissions by secondary mental health service use: A national ecological study [file sj-pdf-1-hsr-10.1177_13558196211036739.pdf]

Online Supplement

Supplement Table S.1. Conditions used to identify potentially avoidable hospital admissions.

Condition	ICD-10 diagnosis codes[2]
Non-specific chest pains	R07.2, 7.3, 7.4
Non-specific abdominal pains	R10
Chronic obstructive pulmonary disease	J40-44
Angina	I20
Minor head injuries	S00
Urinary tract infections	N39.0
Deep vein thrombosis	I80–I82
Epileptic fit	G40-41
Cellulitis	L03
Blocked urinary catheter	T83.0
Hypoglycaemia/diabetic emergencies	E10.0, E11.0, E12.0, E13.0, E14.0, E15, E16.1, E16.2
Falls not elsewhere classified	W00–W19 cause and diagnosis (based on DIAG_01) S00, S10, S20, S30, S40, S50, S60, S70, S80, S90, T00, R

Adapted from Coleman & Nicholl(1)

[2] International Statistical Classification of Diseases and Related Health Problems, Version 10 (ICD-10)(2)

Supplement Table S.2. Predictors of potentially avoidable hospital admissions, description, rationale and data source.

Predictor	Description	Rationale	Source
Socio-demographics and geography			
2015 Index of Multiple Deprivation Domain Scores:<br>Income<br>Employment<br>Education, Skills & Training<br>Crime<br>Barriers to Housing and Services<br>Living Environment	Summary measures of deprivation at CCG-level geography for each of six domains, excluding health domain scores	Deprivation consistently found to predict PAA rates. Robust association between deprivation and mental ill health	GOV.UK[1]
Proportion of population aged over 75 years	Proportion of each CCG population aged over 75 years.	Captures extent of additional demand on health services linked to density of older age in the population	ONS[2]
Proportion of population identifying as Black and minority ethnicities	Proportion of each CCG population identified as non-white British	Ethnicity previously found to be associated with unplanned hospital admissions	ONS[3,4]
Urban/rural	Six-point urban/rural classification scale	Rurality linked to greater distance from services, poorer transport access, lower density of out of hospital support services	ONS[5]
Geographical location	North, Midlands or South England	Inequalities in health between North and South of England	ONS[6]
Underlying morbidity			
QOF prevalence of:<br>Chronic obstructive pulmonary disease (COPD)<br>Diabetes Mellitus (DM)<br>Hypertension (HYP)<br>Serious mental illness (SMI)	2016/2017 QOF data. QOF is a primary care reward and incentive programme records general practice achievement against clinical and organisational targets annually. 'Prevalence' estimates are made for	To capture the underlying burden of mental and physical ill health in the CCG population	NHS Digital[7]

Predictor	Description	Rationale	Source
Depression (DEP)	those recorded with specific conditions against the GP practice list population		
Proportion of population aged 18+ years in contact with mental health services	Average 2016/17 and 2017/18		MHSDS[8]
Proportion of mental health service users treated under the Mental Health Act	Average 2017/18. Proportion of people in contact with secondary mental health services that are formally detained		NHS Digital[9]
Hospital, Emergency Department and ambulance			
Directly age/sex standardised ED attendance rate	Average 2016/17 and 2017/18. Rate of attendance to ED directly standardised to English population	Reflect pressure on/influx into ED department	HES[10]
Median referral to treatment time (weeks)	Average Apr 2017-Mar 2018. Length of time people wait for elective hospital treatment following referral	Reflects access to and demand for elective care	NHS England[11]
Proportion of all unplanned hospital admissions which were referred by GPs	Average 2016/17 and 2017/18. Obtained from referral source	To assess the influence of GP referral behaviour	HES[10]
Proportion of ambulance calls with a face to face response not transported to Type 1 or Type 2 ED (non-conveyance)	Average Apr 2017-Mar 2018. Only available for ambulance services supporting multiple CCGs, reducing between-CCG variation	Reflects the extent to which ambulance staff discharge patients on arrival or take patients to alternative sources of care outside of ED, may also reflect availability of out of hospital services	NHS England[12]
Proportion of 'NHS 111' calls referred to ED	Average Apr 2017-Mar 2018. NHS 111 is a call line intended to provide	Reflects demand on ED which may be avoidable as evidence indicates a large	NHS England[13]

Predictor	Description	Rationale	Source
Proportion of ‘NHS 111’ calls for which an ambulance is despatched	urgent medical support by telephone, and which may direct people away from hospital ED departments to alternative sources of support (if appropriate)	proportion of such referrals could have been treated out of hospital. Increases in ambulance calls direct from NHS 111 have contributed to increasing demand for ambulance services and may lead to a greater number of less urgent callouts, and thus a greater proportion of ambulance calls being discharged at scene (and not conveyed to hospital)	NHS England[13]
Primary care general practice			
Proportion of single-handed GP’s	2017 data. Proportion of GP practices in each CCG that are single handed (have only one GP)	Reflects access to primary care	NHS Digital[14]
Proportion not able to make an appointment to speak to or see someone	2017 GP patient survey data		GP Patient Survey[15]
Proportion able to see GP/nurse within 48 hours	2017 GP patient survey data		
GP's per 100,000 population	2017 data. Number of GPs per 100,000 people in each CCG		NHS Digital[14]
QOF achievement rate	2016/17 QOF data. Single component indicator derived from principal component analysis of QOF quality indicators for COPD, DM, HYP and SMI (see Supplementary Material)	Reflects quality/performance of primary care	NHS Digital[7]
Improving Access to Psychological Therapies (IAPT) access rate	Average 2017/18. Proportion of people identified with depression		NHS Digital[9]

Predictor	Description	Rationale	Source
	and/or anxiety accessing IAPT services	Reflects access to primary care for low level/more commonly experienced mental health problems	
Proportion waiting more than six weeks for IAPT treatment from referral	Average 2017/18. Number of weeks from referral to IAPT to receipt of IAPT treatment, national standards state that 75% of people referred should be seen within six weeks		NHS Digital[9]
Secondary mental health service spending and performance			
Proportion of total core CCG budget allocation spent on mental health services overall	Average 2017/18. Calculated as a proportion of core CCG budget allocation	Due to lack of more specific indicators at CCG-level to assess availability and performance of secondary mental health services, we used spending as a proxy marker of availability of services which may support people out of hospital and prevent PAAs	NHS Digital[9] (Numerator) NHS England[16] (denominator)
Proportion of total core CCG budget allocation spent on early intervention in psychosis (EIP)	Average 2017/18. EIP services work with people who have had a first episode of psychosis. Calculated as a proportion of core CCG budget allocation		
Proportion of total core CCG budget allocation spent on Crisis resolution - home treatment team (CRHT) services	Average 2017/18. CHRT services provide support for people in the community who experience a mental health crisis while out of hospital. Calculated as a proportion of core CCG budget allocation		
Proportion of total core CCG budget allocation spent on ED Liaison services	Average 2017/18. ED Liaison services support ED staff to help patients with mental health needs, as well as educating hospital staff on the needs of patients with poor mental health. Calculated as a		

Predictor	Description	Rationale	Source
	proportion of core CCG budget allocation		
Proportion of mental health service users with a Care Programme Approach (CPA) in place followed up within 7 days of leaving psychiatric hospital	Average 2017/18. CPA is a package of care for people with mental health problems, particularly those with severe conditions and/or at risk of self-harm	Patients on CPA discharged from psychiatric inpatient care should be followed up within 7 days to reduce risk of suicide and social exclusion and to improve care pathways. Reflects link between community and inpatient care.	NHS England[17]
Proportion of people on CPA in employment		Proxy for social isolation, which may increase risk of emergency admission	
Proportion of admissions to psychiatric inpatient wards gate-kept by a CRHT team	Average 2017/18. Proportion of secondary mental health inpatient admissions where alternatives were properly considered beforehand	Gatekeeping prevents unplanned admissions where appropriate	

ONS = Office for National Statistics; NHS = National Health Service; MHSDS = Mental health services dataset; CCG = Clinical Commissioning Group; QOF = Quality and Outcomes Framework; ED = Emergency Department; GP = general practice/practitioner; MH5YFV = Mental Health Five Year Forward View; CPA=Care Programme Approach; CRHT=Crisis resolution home treatment team; PAA=potentially avoidable hospital admission; IAPT=improving access to psychological therapies.
[1]GOV.UK English Indices of Deprivation, https://www.gov.uk/government/statistics/english-indices-of-deprivation-2015 (2015, accessed June 2020). [2]Office for National Statistics (ONS). Annual mid-year population estimates for Clinical Commissioning Groups: Mid-2011, https://www.ons.gov.uk/peoplepopulationandcommunity/populationandmigration/populationestimates/bulletins/annualsmallareapopulationestimates/2013-08-15. (2011, accessed June 2020). [3]ONS. 2011 Census: Key Statistics for Local Authorities in England and Wales, https://www.ons.gov.uk/peoplepopulationandcommunity/populationandmigration/populationestimates/datasets/2011censuskeystatisticsforlocalauthoritiesinenglandandwales. (2011, accessed June 2020). [4]ONS. Lower Layer Super Output Area (2011) to Clinical Commissioning Group to Local Authority District (April 2016) Lookup in England, https://geoportal.statistics.gov.uk/datasets/lower-layer-super-output-area-2011-to-clinical-commissioning-group-to-local-authority-district-april-2016-lookup-in-england/explore (2017, accessed June 2020). [5] ONS. Rural Urban Classification (2011) of CCGs including population in England, https://geoportal.statistics.gov.uk/datasets/d247bded1f4f45ab8c61e05be4303934/explore (2016, accessed June 2020). [6]ONS. Clinical Commissioning Groups (April 2016) Boundaries, https://geoportal.statistics.gov.uk/maps/ons::clinical-commissioning-groups-april-2016-boundaries/about?layer=0 (2016, accessed June 2020). [7] NHS Digital. Quality and Outcomes Framework (QOF) - 2016-17, https://digital.nhs.uk/data-and-information/publications/statistical/quality-and-outcomes-framework-achievement-prevalence-and-exceptions-data/quality-and-outcomes-framework-qof-2016-17 (2017, accessed June 2020). [8] Calculated from MHSDS data requested from NHS Digital. [9]

NHS Digital. Mental Health Five Year Forward View Dashboard (Q1-4), 2016/17 https://www.england.nhs.uk/publication/nhs-mental-health-dashboard/ (2017, accessed June 2020). [10] Calculated from HES Admitted Patient Care and HES Accident & Emergency data requested from NHS Digital. [11] NHS England. Consultant-Led Referral to Treatment Waiting Times, https://www.england.nhs.uk/statistics/statistical-work-areas/rtt-waiting-times/ (2017, accessed June 2020). [12] NHS England. Ambulance Quality Indicators Data 2017/18, https://www.england.nhs.uk/statistics/statistical-work-areas/ambulance-quality-indicators/ambulance-quality-indicators-data-2017-18/ (2018, accessed June 2020). [13] NHS England. NHS 111 Minimum Dataset 2017-18, https://www.england.nhs.uk/statistics/statistical-work-areas/nhs-111-minimum-data-set/nhs-111-minimum-data-set-2017-18/ (2018, accessed June 2020). [14] NHS Digital. NHS Workforce Statistics, September 2017, Provisional Statistics, https://digital.nhs.uk/data-and-information/publications/statistical/nhs-workforce-statistics/nhs-workforce-statistics-september-2017-provisional-statistics (2017, accessed June 2020). [15] NHS England. GP Patient Survey 2017, https://www.england.nhs.uk/statistics/2017/07/06/gp-patient-survey-2017/ (2017, accessed June 2020). [16] NHS England. Allocations for 2016/17 to 2020/21, https://www.england.nhs.uk/allocations/allocations-2016-17-to-2020-21/ (2017, accessed June 2020). [17] Mental Health Community Teams Activity, https://www.england.nhs.uk/statistics/statistical-work-areas/mental-health-community-teams-activity/ (2017, accessed June 2020).

Care Cluster and primary diagnosis data

Identification of mental health service groups

A Care Cluster is part of a currency developed to support the National Tariff Payment System for Mental Health Services.(3) Mental health patients are categorised into one of 21 Care Clusters based on characteristics such as type of disorder (organic, psychotic, non-psychotic) and severity/level of need. Diagnoses likely to belong to each cluster are outlined in NHS guidance. To categorise those in the mental health services dataset (MHSDS) with any 'non-organic' mental health condition we first generated a binary variable to identify patients assigned to any psychotic or non-psychotic cluster (clusters 1-8 or 10-17), coded 1. Organic mental health diagnoses were determined for those assigned to clusters 18-21. If final cluster data were missing for an individual, this was replaced with initial cluster assignment. In total, 46.3% (2016/17) and 48.2% (2017/18) of MHSDS patients were recorded with a valid initial or final care cluster code.

Primary diagnosis code was then examined for those with missing care cluster data (International Statistical Classification of Diseases and Related Health Problems version 10, ICD-10.(2) 18.7% (2016/17) and 20.7% (2017/18) of all MHSDS patients in our sample were assigned a valid primary diagnosis. If available, we coded F00-F09 ICD-10 diagnosis codes as 'organic' conditions. The 'non-organic' conditions group included those whose ICD-10 codes related to both psychotic (F10-F19, F20-29, F30-31, F32.3) and, non-psychotic mental health conditions (F32-33 (excl. F32.3), F40-45, F48, F50, F60).

Supplement Table S.3. Missingness: Socio-demographic characteristics of analysis groups and of those with missing diagnosis/cluster data: frequencies and percentages.

	Comparator[1]		Available diagnosis: organic condition[2]		Available diagnosis: non-organic condition[3]		Percent among all with available diagnosis data	Missing diagnosis	
Sex [a]									
Male	4,895,782	48.9	467,137	44.2	490,883	43.1	43.6	1,595,674	45.0
Female	5,113,738	51.1	590,441	55.8	649,189	56.9	56.4	1,951,766	55.0
Age group (years)[a]									
18-34	1,250,270	12.5	10,753	1.0	287,552	25.2	13.6	479,118	13.5
35-44	727,001	7.3	6,556	0.6	162,401	14.2	7.7	302,109	8.5
45-54	1,023,302	10.2	12,709	1.2	186,107	16.3	9.0	393,914	11.1
55-64	1,313,121	13.1	31,576	3.0	144,290	12.7	8.0	391,408	11.0
65-74	1,942,619	19.4	127,700	12.1	151,682	13.3	12.7	465,947	13.1
75-84	2,166,811	21.7	410,428	38.8	133,603	11.7	24.8	719,805	20.3
85+	1,586,396	15.9	457,856	43.3	74,437	6.5	24.2	795,139	22.4
Deprivation decile (Index of Multiple Deprivation, 2015) [a]									
Least deprived 10%	850,549	8.6	81,093	7.7	52,990	4.7	6.2	230,830	6.6
Less deprived 10-20%	920,100	9.3	93,214	8.9	66,390	5.9	7.3	262,877	7.5
Less deprived 20-30%	945,941	9.5	100,766	9.6	73,163	6.5	8.0	280,219	8.0
Less deprived 30-40%	970,623	9.8	104,518	9.9	84,379	7.5	8.7	301,004	8.6
Less deprived 40-50%	997,059	10.1	112,809	10.7	95,441	8.5	9.6	328,163	9.3
More deprived 10-20%	1,051,161	10.6	111,303	10.6	166,438	14.9	12.8	444,543	12.6
More deprived 20-30%	1,022,514	10.3	117,319	11.1	144,211	12.9	12.0	405,794	11.5
More deprived 30-40%	1,009,807	10.2	112,931	10.7	127,212	11.4	11.0	374,781	10.7
More deprived 40-50%	1,011,997	10.2	111,546	10.6	110,640	9.9	10.2	347,401	9.9
Most deprived 10%	1,134,946	11.5	108,158	10.3	200,019	17.8	14.2	542,295	15.4
Urban/rural [a]									
Urban	7,996,096	80.5	863,278	81.9	971,714	86.7	84.4	2,956,694	83.9
Rural - town and fringe	939,839	9.5	98,891	9.4	81,299	7.3	8.3	296,936	8.4

	Comparator[1]		Available diagnosis: organic condition[2]		Available diagnosis: non-organic condition[3]		Percent among all with available diagnosis data	Missing diagnosis	
Rural – village	703,554	7.1	62,962	6.0	47,455	4.2	5.1	188,098	5.3
Rural – hamlet	300,105	3.0	28,887	2.7	20,944	1.9	2.3	80,899	2.3
Ethnicity [b]									
White British	-	-	534,868	86.5	1,064,343	75.6	78.9	1,288,931	81.7
White Irish	-	-	8,999	1.5	16,984	1.2	1.3	13,476	0.9
White Other	-	-	26,698	4.3	79,357	5.6	5.2	78,246	5.0
Mixed white/Black Caribbean	-	-	1,072	0.2	11,564	0.8	0.6	9,216	0.6
Mixed white/Black African	-	-	356	0.1	3,834	0.3	0.2	2,858	0.2
Mixed white/Asian	-	-	529	0.1	4,890	0.4	0.3	4,195	0.3
Mixed other	-	-	2,200	0.4	12,346	0.9	0.7	13,178	0.8
Indian	-	-	7,521	1.2	21,517	1.5	1.4	24,263	1.5
Pakistani	-	-	4,698	0.8	27,535	2.0	1.6	23,324	1.5
Bangladeshi	-	-	2,029	0.3	10,816	0.8	0.6	6,758	0.4
Other Asian	-	-	4,648	0.8	23,563	1.7	1.4	22,704	1.4
Black Caribbean	-	-	8,269	1.3	30,099	2.1	1.9	16,095	1.0
Black African	-	-	2,820	0.5	25,590	1.8	1.4	15,209	1.0
Other Black	-	-	3,701	0.6	25,310	1.8	1.4	12,863	0.8
Chinese	-	-	895	0.1	3,347	0.2	0.2	3,397	0.2
Other ethnic group	-	-	9,413	1.5	46,286	3.3	2.7	43,417	2.8
Potentially avoidable admissions for physical conditions [a]									
0	5,373,428	81.7	462,010	80.2	643,173	84.6	82.7	1,709,300	80.2
1	1,201,141	18.3	114,263	19.8	116,997	15.4	17.3	422,458	19.8
Total	6,574,569	100.0	576,273	100.0	760,170	100.0	100.0	2,131,758	100.0
Potentially avoidable admission condition type [a]									
Falls	56,534	4.7	18,231	16.0	6,796	5.8	10.8	32,560	7.7
Hypoglycaemic diabetic episode	11,585	1.0	2,499	2.2	1,821	1.6	1.9	6,644	1.6

	Comparator[1]		Available diagnosis: organic condition[2]		Available diagnosis: non-organic condition[3]		Percent among all with available diagnosis data	Missing diagnosis	
Blocked urinary catheter	13,303	1.1	2,481	2.2	1,115	1.0	1.6	6,122	1.4
Cellulitis	101,604	8.5	7,688	6.7	7,246	6.2	6.5	29,373	7.0
Epileptic	19,410	1.6	4,223	3.7	6,263	5.4	4.5	21,616	5.1
Deep vein thrombosis	35,924	3.0	2,040	1.8	2,097	1.8	1.8	9,093	2.2
Urinary tract infection	150,959	12.6	39,618	34.7	15,985	13.7	24.0	80,122	19.0
Minor head injury	6,681	0.6	562	0.5	1,308	1.1	0.8	3,372	0.8
Angina	54,776	4.6	3,023	2.6	3,151	2.7	2.7	12,891	3.1
Chronic obstructive pulmonary disease	162,978	13.6	14,357	12.6	17,788	15.2	13.9	61,652	14.6
Non-specific abdominal pain	264,989	22.1	5,817	5.1	27,753	23.7	14.5	79,663	18.9
Non-specific chest pain	322,398	26.8	13,724	12.0	25,674	21.9	17.0	79,350	18.8
Learning Disabilities flag [b]									
No	-	-	662,976	93.0	1,528,464	97.0	95.7	2,024,378	91.7
Yes	-	-	50,226	7.0	47,692	3.0	4.3	183,585	8.3

MHSDS = Mental health services dataset

[a]From Hospital Episodes Statistics Admitted Patient Care data 2016-2018

[b]From Mental Health Services Dataset patient information 2016-2018

1 Comparator group - records not linked to mental health services dataset over the study period.

2 Organic mental health group - records linked to mental health services dataset over the study period with a primary diagnosis or care cluster assignment linked to any organic mental health condition.

3 Non-organic mental health group - records linked to mental health services dataset over the study period with a primary diagnosis or care cluster assignment linked to any non-organic mental health condition (including psychotic and non-psychotic conditions).

Denominators for the calculation of potentially avoidable admissions

For the ‘comparator’ group the denominator used to calculate rates was the 2016 mid-year CCG population aged 18 or more years, minus the population in contact with mental health services as calculated from MHSDS. The 2016 rather than 2017 mid-year CCG population was used as the mental health services data and other system-wide variables were available for 2016 CCGs (as there were changes to CCGs between the two years involving the merger of a small number of CCG’s into one (e.g. Manchester CCG), to calculate the numerators of these rates we therefore combined admissions data for those merged CCG’s where relevant). For the mental health group, the population aged 18 or more years in contact with mental health services (overall and for organic/non-organic mental health subgroups) in 2017/18 was calculated as a denominator because of unexpectedly low total numbers in contact with services for a small number of CCGs in in 2016/17.

Data preparation

Outliers and winsorisation

Covariates are detailed in Supplementary Table 2. There were outliers on several covariates and outcomes. To avoid potentially influential observations to dominate our statistical models, we winsorised all numeric variables by imposing a minimum equal to the 2.5th percentile of each variable, and a maximum equal to the 97.5th percentile.

Principal component analysis of QOF achievement variables

To reduce the complexity of the data set, we summarised the five quality and outcomes framework (QOF) achievement rate variables by a single principal component, which accounts for 83 % of the variance of these variables. The correlation matrix of the winsorised variables was used to derive the principal component (but the principal component was not in itself winsorised).

Supplement Table S.4. Correlations between five QOF achievement variables among 207 CCGs.

	Hypertension	COPD	Diabetes	Mental Health
COPD	0.84			
Diabetes	0.75	0.80		
Mental Health	0.81	0.86	0.81	
Depression	0.65	0.75	0.72	0.81

COPD = chronic obstructive pulmonary disease

Correlations among the unwinsorised variables were virtually identical. Given that the correlations were high, dimension reduction via a principal component was deemed suitable and advantageous for the analysis as a whole. The principal components analysis suggested that a single component accounts for 83 % of the variance in the five variables, and that further components account for very little.

Supplement Table S.5. Results of a principal components analysis on five QOF achievement variables among 207 CCGs.

	Principal Components				
	1	2	3	4	5
Proportion of variance	0.825	0.073	0.048	0.028	0.026
Standardised loadings					
Hypertension	0.89				
COPD	0.94				
Diabetes	0.90				
Mental Health	0.95				
Depression	0.86				

COPD= chronic obstructive pulmonary disease

Categorical variables

We dealt with categorical covariates as follows: we dichotomised the six-category rural-urban classification (RUC11CD) into a dummy variable indicating "predominantly urban (categories 4-6), with "rural or significant rural" (categories 1-3) serving as the reference category. CCG region was recoded into two dummy variables: "north" and "south", with midlands as the reference category.

Standardisation

We standardised all numeric variables to have a mean of zero and a standard deviation of 1. For the purpose of the MIRL estimation (see below), the dummy variables for urbanity and region were also standardised.

Transformations: fractional polynomials

Exploratory analysis used fractional polynomials(4) to identify transformations of covariates that would improve the fit in case of potential non-linear relationships with the outcome. Across 38 covariates and 3 outcomes, only one transformation was suggested by the fractional polynomial analysis (using the criterion $p < 0.01$). This suggested an extremely unlikely shape for the association and did not fit the data better than a simple linear prediction. Thus, no transformations were used in the analysis.

Missing values and the multiple imputation random lasso (MIRL)

Seven of our 38 predictor variables had missing values. The maximum number of missing values for one predictor was 15. The overall number of missing values was 39 (0.5 % of all covariate values). Of the 207 CCGs, 180 had complete data. The remaining 27 CCGs had between one and four missing values. Thus, although the extent of missingness is small, a complete cases analysis would have considerably reduced our analysis data set, and would likely have led to bias, since the assumption of Missing Completely At Random is unlikely to hold.

We employed multiple imputation of missing values by chained equations.(5) All predictors, primary outcomes, and four auxiliary variables were included in the imputation model. No predictors were transformed. Values were imputed by predictive mean matching, to avoid

the risk of creating outlier values. Due to the low rate of missing values overall, five imputed data sets were deemed sufficient. Model selection in the presence of missing data is an area of active research.(6) In our study, we needed to consider in particular how to combine lasso estimates from several imputed data sets. We chose the multiple imputation random lasso (MIRL)(7) approach to do this, and report the final MIRL estimates. The MIRL stages followed were:

(1) Multiple imputation. Use multiple imputation by chained equations to produce m imputed data sets (m = 5 in our case). Standardise all covariates.
(2) Bootstrapping and calculating variable importance. From each imputed data set, create b bootstrap samples (we used b = 40). For each of the resulting m×b bootstrap samples, apply the lasso-OLS estimator(8, 9) to obtain coefficient estimates. We used 10-fold crossvalidation to select the tuning parameter lambda. The average of the m×b coefficients for each covariate is called that covariate's importance.
(3) Calculate initial MIRL estimates. For each of the m×b samples, randomly select half the covariates, with selection probability proportional to the covariate's importance. Apply lasso-OLS to each of the resulting reduced data sets and average each covariate's coefficients to obtain the initial MIRL estimate. If a variable is not present in a data set, its coefficient from that data set is taken to be zero.
(4) Choose a stable variable set and obtain final MIRL estimates. Calculate each variable's empirical selection probability as the proportion of times its lasso-OLS estimate in Step 3 was non-zero.
Variables with selection probability higher than a probability threshold π_{thr} form the stable variable set. Their final MIRL estimates are equal to their initial MIRL estimates. All variables outside of the stable variable set have final MIRL estimates equal to zero. π_{thr} is chosen by stability selection.(10)

Sensitivity analyses

Lasso regression of avoidable admission rates on Clinical Commissioning Group characteristics: final MIRL estimates Secondary mental health services users with non-organic mental health conditions[1]. Sensitivity analysis including CCGs with at least 50% of available cluster/diagnosis data (n=127) in the Mental Health Services Dataset (MHSDS).

Predictor	Estimated standardised coefficient	Bootstrap quantiles: (2.5 %, 97.5 %)
Urban	0.140	(-0.033, 0.404)

Note: All coefficients are xy-standardised. Bootstrap quantiles represent the quantiles of the coefficient distribution generated by bootstrapping in the final stage of MIRL. They are not confidence intervals in the strict sense and are given for informal illustration only.
MIRL=multiple imputation random lasso
[1] Records linked to mental health services dataset over the study period with a primary diagnosis or care cluster assignment linked to any non-organic mental health condition (including psychotic and non-psychotic conditions

Lasso regression of avoidable admission rates on Clinical Commissioning Group characteristics: final MIRL estimates for Secondary mental health services users with non-organic mental health conditions[1]. Sensitivity analysis including CCGs with at least 30% of available cluster/diagnosis data (n=180) in the Mental Health Services Dataset (MHSDS).

Predictor	Estimated standardised coefficient	Bootstrap quantiles: (2.5 %, 97.5 %)
COPD prevalence	0.693	(0.197, 1.118)
Population over 75 years (%)	-0.200	(-0.555, 0.093)
Adult population in contact with secondary mental health services (%)	-0.360	(-0.661, 0)
Receipt IAPT treatment within 6 weeks (%)	0.179	(0, 0.317)
Urban	0.123	(0, 0.324)

Note: All coefficients are xy-standardised. Bootstrap quantiles represent the quantiles of the coefficient distribution generated by bootstrapping in the final stage of MIRL. They are not confidence intervals in the strict sense and are given for informal illustration only.
MIRL=multiple imputation random lasso
[1] Records linked to mental health services dataset over the study period with a primary diagnosis or care cluster assignment linked to any non-organic mental health condition (including psychotic and non-psychotic conditions

References

1. Coleman P, Nicholl J. Consensus methods to identify a set of potential performance indicators for systems of emergency and urgent care. J Health Serv Res Policy 2010 Apr;15(2_suppl):12-8.
2. World Health Organization. International Statistical Classification of Diseases and Related Health Problems 10th Revision (ICD-10) Version, https://icd.who.int/browse10/2016/en (2016, accessed June 2020).
3. NHS England. Mental Health Clustering Booklet (v5.0) (2016/17), https://assets.publishing.service.gov.uk/government/uploads/system/uploads/attachment_data/file/499475/Annex_B4_Mental_health_clustering_booklet.pdf (2016, accessed June 2020).
4. Sauerbrei W, Royston P. Building multivariable prognostic and diagnostic models: transformation of the predictors by using fractional polynomials. J Roy Stat Soc: Series A (Statistics in Society) 1999;162(1):71-94.
5. Carpenter J, Kenward M. Multiple imputation and its application. West Sussex: John Wiley & Sons; 2012.
6. Zhao Y, Long Q. Variable selection in the presence of missing data: imputation-based methods. WIREs Computational Statistics 2017 Sep;9(5):e1402.
7. Liu Y, Wang Y, Feng Y, Wall MM. Variable selection and prediction with incomplete high-dimensional data. Ann Appl Stat 2016 Mar;10(1):418.
8. Belloni A, Chernozhukov V. Least squares after model selection in high-dimensional sparse models. Bernoulli 2013;19(2):521-47.
9. Efron B, Hastie T, Johnstone I, Tibshirani R. Least angle regression. Ann Stat. 2004;32(2):407-99.
10. Meinshausen N, Bühlmann P. Stability selection. J Roy Stat Soc: Series B (Statistical Methodology) 2010 Sep;72(4):417-73.
